# Study on the expression of c-Met in gastric cancer and its correlation with preoperative serum tumor markers and prognosis

**DOI:** 10.1186/s12957-022-02659-2

**Published:** 2022-06-16

**Authors:** Zhengchao Zhang, Lele Miao, Song Wang, Yang Zhao, Yongqiang Xie, Heng Yun, Zhijian Ren, Guan Wang, Muzhou Teng, Yumin Li

**Affiliations:** 1 Department of General Surgery, Lanzhou University Second Hospital, Lanzhou, 730000 China; 2 Key Laboratory of Digestive System Tumors of Gansu Province, Lanzhou, 730000 China; 3 Department of General Surgery, The Third Affiliated Hospital of Gansu University of Traditional Chinese Medicine, Lanzhou, 730900 China

**Keywords:** c-Met, Gastric cancer, CA125, AFP, Prognosis, Independent risk factors

## Abstract

**Background:**

Studies have found that c-Met plays a critical role in the progression of solid tumors. This study aimed to investigate the expression of c-Met in gastric cancer (GC) and its correlation with preoperative serum tumor markers and prognosis, in order to provide a more theoretical basis for targeting c-Met in the treatment of GC.

**Methods:**

Ninety-seven patients who underwent curative gastrectomy in our hospital from December 2013 to September 2015 were included in this study. The tissue microarray was constructed by paraffin-embedded tumor tissue of enrolled patients, including 97 GC points and 83 paracancerous points. Then, it was used for c-Met immunohistochemical staining, followed by an immunological *H*-score. The clinical baseline data and 5-year survival of patients with low and high c-Met expression were compared. Besides, the correlation between the expression of c-Met in tumor tissues and preoperative serum tumor markers was investigated. Finally, multivariate Cox regression analysis was used to explore the survival risk factors of patients.

**Results:**

c-Met has a high expression rate in GC tissues 64.95% (63/97). The expression of c-Met was significantly different in different clinicopathological stages (*p* < 0.05); the high expression group also had a higher M stage and clinicopathological stage of GC. The correlation test between the c-Met *H*-score and CA125 was statistically significant (*p* = 0.004), indicating a positive correlation. Furthermore, high c-Met expression correlated with poor overall survival (OS) for 5 years (*p* = 0.005). It was also found that the high expression of c-Met in stage I–II patients was correlative with poor OS for 5 years (*p* = 0.026), while stage III–IV patients had no statistical significance (*p* > 0.05). Multivariate Cox regression analysis showed that c-Met might be an independent risk factor for survival 5 years after surgery.

**Conclusion:**

This study found that the high expression of c-Met in GC tissues was associated with poor 5-year OS in GC patients and was an independent risk factor for 5-year survival after curative gastrectomy. The expression of c-Met in GC tissues was also positively correlated with preoperative serum CA125.

## Introduction

Gastric cancer (GC) is one of the most common digestive system malignancies globally [1]. It is the fifth most common cancer and fourth most common cause of cancer death worldwide, with more than 1,000,000 new cases and 769,000 deaths due to GC in 2020 [2]. Unfortunately, most patients are diagnosed in the middle and late stages, so the survival rate after surgery is meager, and some patients lose the opportunity for surgery. The 5-year survival rate for GC is 31% in the USA, 19% in the UK, and 28% in China [3]. Consequently, there is an urgent need for effective therapeutic methods to treat these patients and improve clinical outcomes.

c-mesenchymal-epithelial transformation factor (c-Met) is involved in the tumorigenesis of various cancers, including GC [4]. c-Met inhibitors have attracted much attention due to their antitumor activity in various solid tumors. In recent years, inhibitors and mAb of c-Met have not achieved significant efficacy in clinical studies of GC [5–9]. However, MET proto-oncogenes do participate in the progression of various solid tumors and mediate the proliferation and metastasis of various tumor cells [10]. Therefore, it is necessary to study further the expression of c-Met in GC and its role in the tumor progression to provide the theoretical basis for optimizing targeted c-Met therapy in GC to improve the survival of patients.

Serum tumor markers such as AFP, CEA, CA-199, CA-125, CA-153, and CA-50 have been applied in cancer diagnosis and monitoring. Studies have found that the levels of some peripheral serum tumor markers are correlated with the prognosis of GC patients, so these tumor markers can be used as predictors of tumor progression in GC patients [11, 12]. Nonetheless, most of the studies mainly aimed to investigate the influence of preoperative and postoperative serum tumor marker levels on the clinical prognosis of patients [13–15]. There are few studies on the correlation between tumor markers and carcinogenic factors. If there is a correlation between them, peripheral serum tumor marker monitoring can better guide clinicians to use targeted therapy to develop personalized treatment strategies. At the same time, it will also bring more convenient GC monitoring services and a more accurate medical treatment experience for patients.

Based on the expression of c-Met in GC tissues, the relationship between c-Met and the clinical prognosis was researched in this study. Furthermore, the correlation between c-Met and serum tumor markers (AFP, CEA, CA199, CA153, CA125, CA50) in GC progression was discussed. Finally, the risk factors for postoperative survival of GC patients were examined. In order to provide a more theoretical basis for the targeted c-Met treatment of GC.

## Materials and methods

### Patients and samples

This study included 97 patients who underwent curative gastrectomy in our hospital from December 2013 to September 2015, and the included patients had complete clinicopathological data and 5-year postoperative follow-up records. Clinicopathological data included age, sex, operation date, operation method, tumor location, tumor size (maximum diameter), TNM stage, degree of tumor differentiation, *Helicobacter pylori* (*H. pylori*) infection, HER2 expression in GC tissues, and preoperative peripheral serum tumor marker levels (AFP, CEA, CA-199, CA-125, CA-153, CA-50), which were obtained from medical records. The 7th edition of the AJCC TNM staging system was used for pathological TNM staging, and clinicopathological staging corresponding to TNM staging of GC was used (7th edition of NCCN, 2010).

The study was approved by the Ethics Committee of Lanzhou University Second Clinical Medical School and is consistent with the Declaration of Helsinki. The research group informed each patient of the significance of this study and signed a written consent form. Patients to be included in the study must meet the following inclusion criteria: (1) curative gastrectomy, (2) postoperative pathological diagnosis was gastric adenocarcinoma, and (3) postoperative paraffin tissue specimens are available for study. The exclusion criteria were emergency surgery, palliative surgery for GC, patients with other tumors, patients who had not received oxaliplatin plus tigio after surgery or who had less than 6 courses, and patients who had received other chemotherapy regimens after surgery.

### Detection method

HE and immunohistochemical staining confirmed postoperative specimens on the pathological test platform of our hospital. HER2 expression data was obtained by immunohistochemical staining. A preoperative C14 breath test diagnosed *H. pylori* infection. Five milliliters of fasting venous blood was collected from the patient before surgery, and the serum was separated after centrifugation. Roche Cobas8000 chemiluminescence immunoassay was used to detect tumor markers in the serum. All tumor marker kits are provided by Roche and are tested strictly in accordance with the kit instructions.

### Tissue microarray construction

Paraffin-embedded tumor tissues were collected to prepare tissue microarray. HE-stained sections were first reviewed, and representative tumor regions were selected. Then, a drilling machine was used to drill holes on the marked blank wax block target, and an automatic tissue microarray instrument (Jinan Tangier Electronics Co., LTD.) was used to drill tissue cores (diameter 1.5 mm) from the target paraffin block and transfer the tissue cores into the marked blank wax block holes. Finally, the tissue microarray wax block was sliced by a microtome (thickness 4 μm), and then the slice was transferred to the slide to make the tissue microarray. One tumor tissue and one paracancerous tissue were taken from each case, and a total of 97 cases of gastric adenocarcinoma were included in this tissue microarray, including 97 cancer points and 83 paracancerous points.

### Immunohistochemistry

The expression of c-Met in GC tissues was detected by immunohistochemistry. c-Met rabbit monoclonal antibody (Cat: AB51067) was purchased from Abcam, Inc. The tissue microarray was heated in an oven for 1 h for conventional dewaxing, followed by xylene dewaxing and hydration in graded ethanol. Subsequently, thermally induced antigen repair was performed with citric acid buffer and cooled naturally to room temperature. The tissue microarray was incubated at room temperature with 3% hydrogen peroxide for 20 min to block endogenous peroxidase activity. The tissue microarray was then sealed with 1–2% goat serum for 30min and was incubated overnight with primary antibody (the primary antibody concentration was 1:200 in the preliminary experiment) at 4 °C. The tissue microarray was incubated with 1:50 diluted goat anti-rabbit IgG secondary antibody at RT for 60 min on the next day. DAB color solution was added to the slices to make the color. Finally, the slice was restained with hematoxylin, then dehydrated and sealed.

### Immunohistochemical scores

The immunomicroarray was read and analyzed under an Olympus microscope (CX31-LV320). Tumor c-Met expression was evaluated according to the immunological histochemistry score (*H*-score) system, with *H*-score = staining intensity × staining area grade. The staining intensity was divided into 0 (no staining), 1+ (weak staining), 2+ (medium staining), and 3+ (strong staining). The staining area was classified as 0 (no cell staining), 1+ < 25%, 25% ≤ 2+ < 50%, and 3+ ≥ 50%. In this study, *H*-score < 3 was defined as a weak expression, and *H*-score ≥ 3 was defined as a high expression. Immunohistochemical staining assessment was performed by two chief pathologists blinded to the clinicopathological diagnosis of the patient.

### Statistical analysis

The SPSS 20.0 software was used for statistical analysis. Categorical data is represented by number (%). The measurement data were tested for normality by the Shapiro-Wilk test, and the mean (SD) was used to represent the data with normal distribution. The chi-square test was used to evaluate the categorical data. The Student *t*-tests were used to evaluate the measurement data. The Pearson correlation test evaluated the correlation between c-Met expression and blood tumor markers, and a two-sided test was used. The 5-year overall survival (OS) curve and univariate survival analysis were compared using Kaplan-Meier curves (log-rank test). OS is defined as counting from the date of surgery to the date of death from any cause of death. In univariate survival analysis, AFP, CEA, CA199, CA153, CA125, and CA50 were converted into categorical variables using the median as a truncation value. Multivariate analyses were performed to analyze the survival risk factors by Cox regression. The hazard ratio (HR) and corresponding 95% confidence interval (CI) were calculated. All *p* < 0.05 were considered statistically significant.

## Results

### Clinicopathologic demographics of the GC patients

There were 23 female patients (23.7%) and 74 male patients (76.3%) in the study. The mean age was 59.0 (10.0) years. According to different tumor locations, included GC patients were divided into gastric body 14(14.43%), gastric antrum 68(70.10%), and fundus 15 (15.46%). The patients of clinicopathological stages I–II were 47 (48.45%) and III–IV 50 (51.55%). All patients underwent curative gastrectomy and lymph node dissection, and the scope of lymph node dissection was determined according to the 2010 Japanese guidelines for the treatment of GC [16]. All patients received 6–8 cycles of chemotherapy that oxaliplatin combined with tegio after surgery.

### Comparative analysis of c-Met expression in GC and paracancerous tissues

**Fig. 1:**
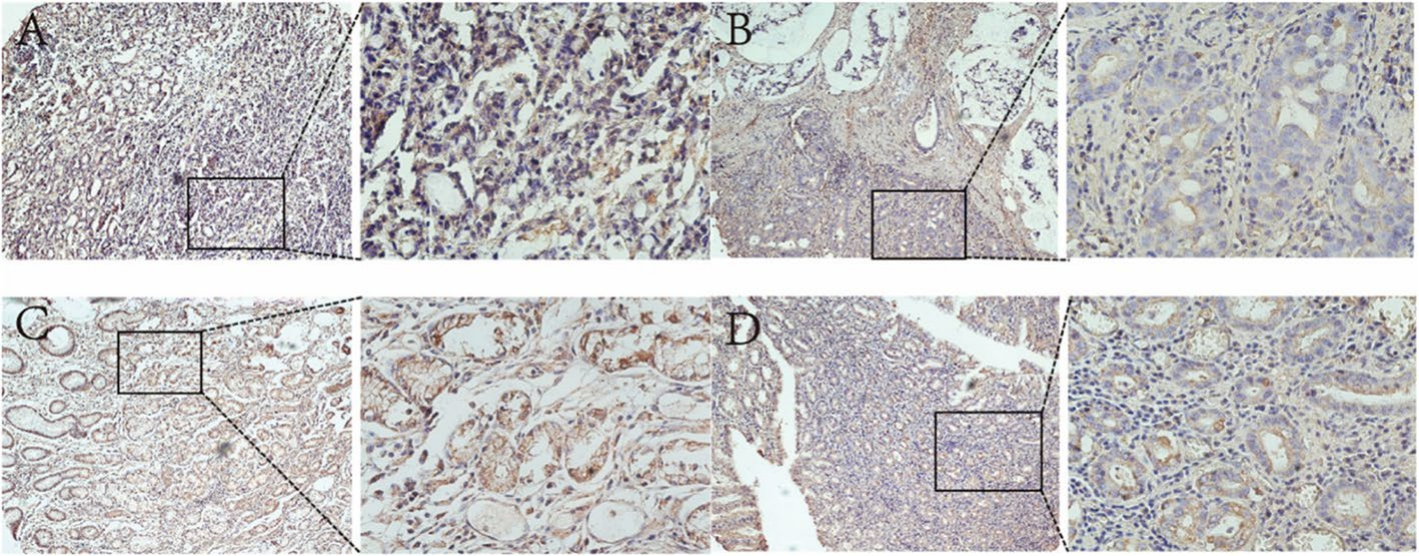
Representative immunohistochemical images of c-Met expression in GC and paracancerous tissues. **A** High expression of c-Met in GC tissues. **B** Low expression of c-Met in GC tissues. **C** High expression of c-Met in paracancerous tissues. **D** Low expression of c-Met in paracancerous tissues. The scale bars are 100 μm and 20 μm, respectively

**Fig. 2:**
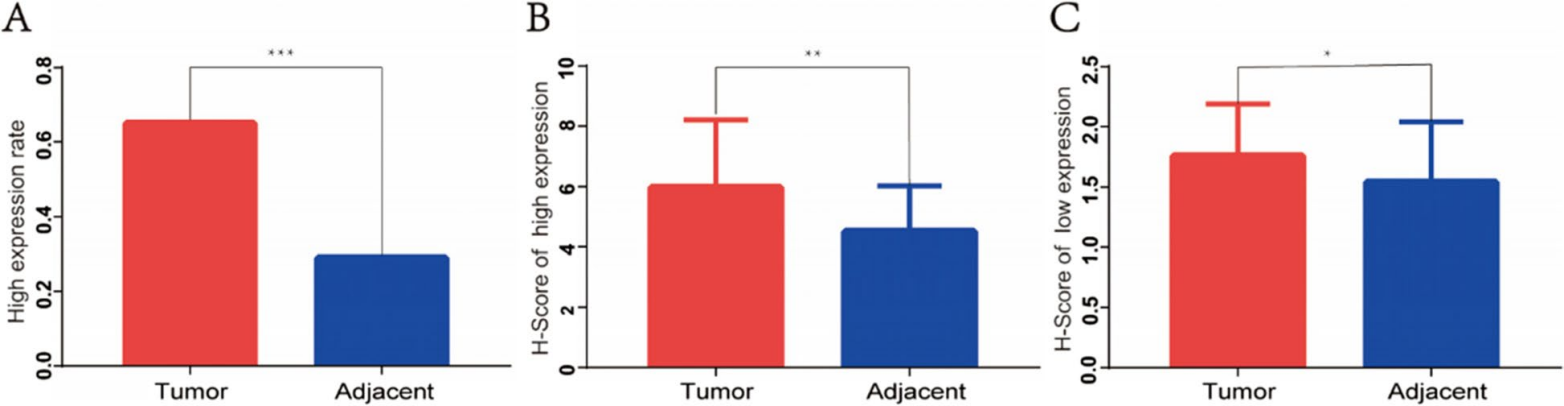
Comparison of c-Met high expression rate and *H*-score in cancer and paracancerous tissues. **A** Comparison of c-Met high expression rate between GC tissues and paracancerous tissues. **B** Comparison of *H*-score between GC tissues and paracancerous tissues with c-Met high expression. **C** Comparison of *H*-score between GC tissues and paracancerous tissues with c-Met low expression

c-Met was stained in all cell membranes, and some cells were stained in the inner membrane (Fig. 1). The overexpression rate of GC tissues was 64.95% (63/97) and that of paracancerous tissues was 28.92% (24/83) (Table 1). The expression of c-Met in GC tissues was significantly higher than that in paracancerous tissues (*p* < 0.001) (Fig. 2A). There were statistical differences in the *H*-score between GC tissues and paracancerous tissues in patients with high and low expression of c-Met (*p* = 0.004, *p* = 0.033) (Fig. 2B, C). The mean *H*-score expression of c-Met in cancer tissues was higher than that in paracancerous tissues in patients with high and low expression of c-Met.

**Table 1:** Comparative analysis of c-Met expression in GC and paracancerous tissues

Variable	Tumor (97)	Paracancerous (83)	*t*/*X*^2^	*p*-value
Low expression group, *n* (%)	34 (35.05)	59 (71.08)	23.255	< 0.001
High expression group, *n* (%)	63 (64.95)	24 (28.92)		
*H*-score of low expression group, mean (SD)	1.76 (0.43)	1.54 (0.50)	2.162	0.033
*H*-score of high expression group, mean (SD)	5.97 (2.24)	4.50 (1.53)	2.958	0.004

### Comparative analysis of clinical baseline data between c-Met low expression and high expression group

In the comparison of clinical baseline data between c-Met low expression and high expression groups (age, sex, tumor size, tumor location, TNM stage, clinicopathological stage, degree of differentiation, positive expression of HER2, and *H. pylori*), there was an apparent difference in the M stage and clinicopathological stage (*p* < 0.05) (Table 2). Compared with the low expression group, the high expression of c-Met was associated with a greater likelihood of tumor metastasis and a higher clinicopathological stage.

**Table 2:** Comparative analysis of clinical baseline data between the c-Met low expression and high expression groups

Variable	c-Met expression [*n* (%)/mean (SD)]		*t*/*X*^2^	*p*-value
	c-Met low group (*n* = 34)	c-Met high group (*n* = 63)		
Age (years)	57.35 (10.63)	59.95 (9.61)	1.224	0.224
Sex				
Female	11 (32.35)	12 (19.05)	2.161	0.142
Male	23 (67.65)	51 (80.95)		
Tumor diameter (cm)	3.74 (1.82)	4.11 (1.75)	1.001	0.319
Tumor location				
Gastric body	5 (14.71)	9 (14.29)	0.554	0.758
Gastric antrum	25 (73.53)	43 (68.25)		
Gastric fundus	4 (11.76)	11 (17.46)		
T stage				
T (1, 2)	13 (38.24)	13 (20.63)	3.487	0.062
T (3, 4)	21 (61.76)	50 (79.37)		
N stage				
N−	11 (32.35)	16 (25.40)	0.532	0.466
N+	23 (67.65)	47 (74.60)		
M stage				
M−	33 (97.06)	51 (80.95)	4.936	0.022
M+	1 (2.94)	12 (19.05)		
Clinicopathologic stage				
I–II	22 (64.71)	25 (39.68)	5.536	0.019
III–IV	12 (35.29)	38 (60.32)		
Differentiated degree				
Poor differentiation	14 (41.18)	40 (63.49)	4.908	0.086
Moderate differentiation	17 (50.00)	21 (33.33)		
High differentiation	3 (8.82)	2 (3.17)		
HER2-IHC				
Missing	1 (2.94)	1 (1.59)		
Negative	31 (91.18)	56 (88.89)	0.365	0.546
Positive	2 (5.88)	6 (9.52)		
*H. pylori*				
Missing	6 (17.65)	17 (26.98)		
Negative	14 (41.18)	23 (36.51)	0.001	1.000
Positive	14 (41.18)	23 (36.51)		

### The correlation test between c-Met and serum tumor markers

The Pearson test was performed with the *H*-score of c-Met in GC tissues and serum tumor markers (AFP, CEA, CA199, CA153, CA125, and CA50) related to the digestive system. The study found that the correlation between c-Met and preoperative serum CA125 level of patients was statistically significant (*p* = 0.004) (Table 3), showing a positive correlation. The expression of c-Met increased with the increase of CA125.

**Table 3:** Correlation test between C-MET and serum tumor markers

Variable	Total (*n*)	Value [mean (SD)]	*r*	*p*-value
AFP (ng/ml)	78	43.96 (118.05)	0.043	0765
c-MET (*H*-score)	78	5.70 (2.21)		
CEA (ng/ml)	78	48.91 (118.88)	0.020	0.890
c-MET (*H*-score)	78	5.70 (2.21)		
CA199 (μ/ml)	78	51.87 (115.62)	0.042	0.773
c-MET (*H*-score)	78	5.70 (2.21)		
CA153 (μ/ml)	77	11.71 (10.52)	0.131	0.370
c-MET (*H*-score)	77	5.73 (2.22)		
CA125 (μ/ml)	77	13.66 (34.33)	0.322	0.004
c-MET (*H*-score)	77	4.32 (2.60)		
CA50 (μ/ml)	75	11.96 (24.01)	0.052	0.658
c-MET (*H*-score)	75	4.37 (2.61)		

### The 5-year survival analysis of patients with high and low expression of c-Met

**Fig. 3:**
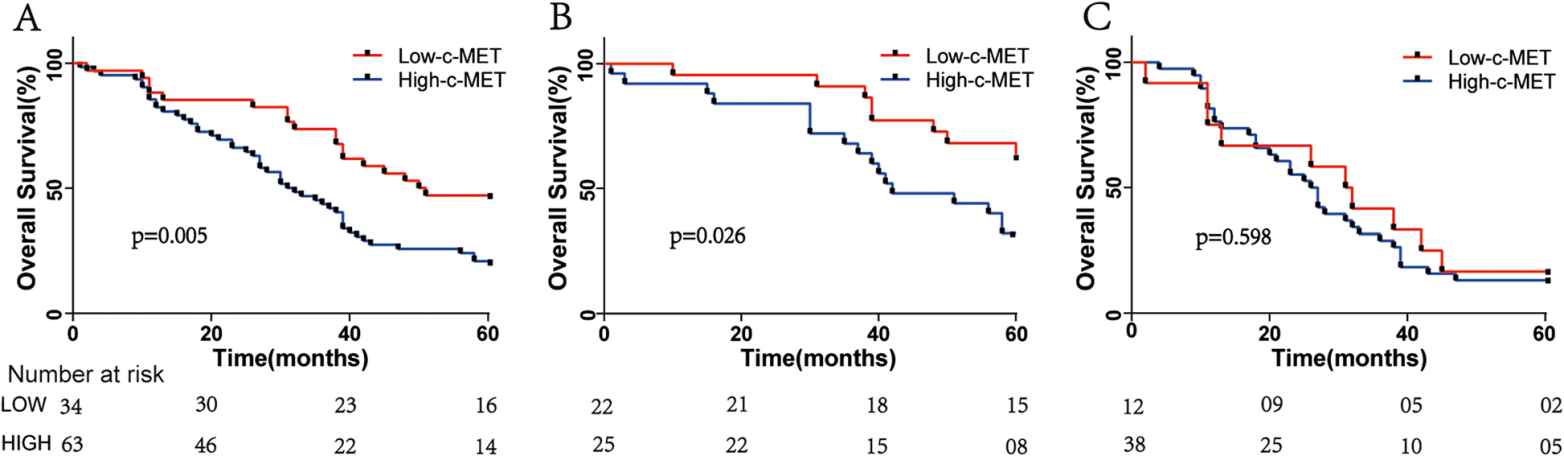
Kaplan-Meier survival curves of log-rank test for 5-year OS. **A**–**C** Five-year OS curves of patients with high and low expression of c-Met (totality, stage I–II patients, and stage III–IV patients)

The 5-year OS curves of patients with high and low c-Met expression showed statistically significant differences (95% CI 1.298–3.409; *p* = 0.005) (Fig. 3A), and the median survival time was 31.5 months and 50.5 months, respectively. The 5-year OS of the c-Met high expression group was significantly worse than those of the low expression group. Patients were grouped according to the different clinicopathological stages of the tumor, and then 5-year OS curves were constructed. The results also showed statistically significant differences in stages I–II (95% CI 1.132–5.458; *p* = 0.026) (Fig. 3B), and the median survival time was undefined months and 42 months, respectively. The 5-year OS in the c-Met high expression group was significantly lower than that in the low expression group. There was no significant difference in the 5-year OS between the two groups of patients in stages III–IV (*p* > 0.05) (Fig. 3C).

### Univariate analysis of 5-year survival

The log-rank test was used for the 5-year univariate survival analysis. The results showed that age, tumor size, clinicopathological stage, c-Met expression, and serum AFP level were correlated with 5-year survival risk (*p* < 0.05) (Table 4). Patients with older age, larger tumor diameter, higher clinicopathologic staging, higher c-Met expression, and higher AFP levels were associated with a greater survival risk of fewer than 5 years. Univariate survival analysis of other pathological related factors and 5-year survival showed no statistical significance (*p* > 0.05).

**Table 4:** Univariate analysis of 5-year survival

Variable		Univariable survival analysis [*n* (%)]			
		Survival (*n* = 30)	Death (*n* = 67)	*X*^2^	*p*-value
Age	< 60	19 (63.33)	30 (44.78)	4.912	0.027
	≥ 60	11 (36.67)	37 (55.22)		
Sex	Male	24 (80.00)	50 (74.63)	0.056	0.813
	Female	6 (20.00)	17 (25.37)		
Tumor diameter	< 4.0	19 (63.33)	29 (43.28)	6.681	0.009
	≥ 4.0	11 (36.67)	38 (56.72)		
Tumor location	Gastric body	3 (10.00)	11 (16.42)	2.503	0.286
	Gastric antrum	20 (66.67)	48 (71.64)		
	Gastric fundus	7 (23.33)	8 (11.94)		
Operation method	Distal gastrectomy	20 (66.67)	46 (68.66)	1.81	0.405
	Proximal gastrectomy	6 (20.00)	10 (14.93)		
	Total gastrectomy	4 (13.33)	11 (16.42)		
Clinicopathologic stage	I–II	23 (76.67)	24 (35.82)	22.32	< 0.001
	III–IV	7 (23.33)	43 (64.18)		
Differentiated degree	Poor differentiation	13 (43.33)	41 (61.19)	3.902	0.142
	Moderate differentiation	15 (50.00)	23 (34.33)		
	High differentiation	2 (6.67)	3 (4.48)		
c-Met expression	Low expression	16 (53.33)	18 (26.87)	7.929	0.005
	High expression	14 (46.67)	49 (73.13)		
HER2-IHC	Negative	29 (96.67)	58 (89.23)	1.913	0.167
	Positive	1 (3.33)	7 (10.77)		
AFP (ng/ml)	< 2.54	7 (28.00)	17 (32.08)	8.92	0.002
	≥ 2.54	18 (72.00)	36 (67.92)		
CEA (ng/ml)	< 2.13	11 (44.00)	28 (52.83)	0.845	0.358
	≥ 2.13	14 (56.00)	25 (47.17)		
CA199 (μ/ml)	< 12.78	13 (52.00)	15 (28.30)	0.448	0.503
	≥ 12.78	12 (48.00)	38 (71.70)		
CA153 (μ/ml)	< 7.70	15 (60.00)	23 (44.23)	1.023	0.312
	≥ 7.70	10 (40.00)	29 (55.77)		
CA125 (μ/ml)	< 6.80	13 (54.17)	25 (47.17)	0.055	0.814
	≥ 6.80	11 (45.83)	28 (52.83)		
CA50 (μ/ml)	< 4.53	12 (50.00)	25 (49.02)	0.137	0.711
	≥ 4.53	12 (50.00)	26 (50.98)		
*H. pylori*	Negative	10 (45.45)	27 (51.92)	0.427	0.514
	Positive	12 (54.55)	25 (48.08)		

### Multivariate Cox regression analysis of 5-year survival

Multivariate Cox regression analysis was performed for the indexes with statistical significance in univariate survival analysis. The results showed that age, clinicopathological stage, high expression of c-Met, and preoperative serum AFP might be independent risk factors for survival 5 years after surgery (*p* < 0.01) (Table 5). Patients with older age, higher clinicopathological stage, higher c-Met expression, and higher AFP levels were at a greater survival risk of fewer than 5 years.

**Table 5:** Multivariate Cox regression analysis of 5-year survival

5-year overall survival variable	*B*	S.E	Wals	*p*-value	HR	95% CI
Age	0.054	0.017	10.252	0.001	1.055	1.021–1.091
Clinicopathologic stage	1.139	0.309	13.630	< 0.001	3.124	1.706–5.719
c-Met expression	0.192	0.055	12.298	< 0.001	1.211	1.088–1.348
AFP	0.015	0.004	14.346	< 0.001	1.015	1.007–1.023

## Discussion

In this study, immunohistochemistry confirmed that the high expression rate of c-Met in GC tissues was significantly higher than that in paracancerous tissues (64.95% vs 28.92%). Secondly, the study also found that the mean *H*-score expression of c-Met in patients with high and low expression of c-Met in cancer tissues was higher than that in paracancerous tissues (*p* < 0.05). It indicates that c-Met is highly expressed in most GCs and exerts a vital function. Many studies have also confirmed that c-Met promotes the development, proliferation, invasion, metastasis, and angiogenesis of solid tumor cells through downstream signaling pathways and even chemotherapy resistance [17–20]. This study also found that the high expression of c-Met was associated with poor postoperative survival and was positively correlated with the preoperative serum CA125 level of patients, suggesting that c-Met is a promising molecule in the treatment of GC, which can be used for targeted therapy and also conducive to the monitoring of tumor progression.

c-Met-targeted therapy in GC mainly includes tyrosine kinase inhibitors, monoclonal antibodies, and c-Met-targeted adoptive immunotherapy. Tyrosine kinase inhibitors and monoclonal antibodies have shown obvious antitumor activity in cell and xenograft tumor models [21–25], while most tumors have not achieved prominent antitumor activity in clinical trials [6, 26]. Only a few tumors have shown encouraging antitumor activity, especially in the treatment of NSCLC (non-small cell lung cancer) [27, 28]. Furthermore, c-Met-targeted CAR-T cells have shown good antitumor activity in preclinical studies of GC [29, 30]. Since adoptive immunotherapy mainly relies on the particular expression of c-Met on the cell membrane of GC, it is not limited to the carcinogenic mechanism of c-Met. This study also found that c-Met expression was significantly increased in high-grade clinicopathological stages of GC. Therefore, the above indicated that targeted c-Met adoptive immunotherapy in the middle and late stages of GC might be a new direction for the treatment. Currently, two clinical studies on c-Met CAR-T cells in the treatment of liver cancer, GC, and other solid tumors of the digestive system are being implemented in China (NCT03672305, NCT03638206) to evaluate the efficacy and safety of c-Met CAR-T cells in solid tumors of the digestive system and expect to achieve good results.

Xie et al. [31] found that c-Met expression was significantly increased in GC specimens with *H. pylori* infection, and in vitro experiments also confirmed that *H. pylori* infection may activate the HGF/c-MET signaling pathway, which may be involved in the occurrence of GC. Secondly, Huang et al .[32] carried out an in-depth study and found that c-Met expression increased significantly in GC tissues with positive cytotoxin-related gene A (CagA) and *H. pylori* infection. Meanwhile, it was also found that the activation of the c-Met signaling pathway was associated with inhibiting autophagy and promoting tumor cell invasion and metastasis in patients. However, our study found no significant increase in c-Met expression in the *H. pylori*-positive group. *H. pylori* infection is only one of the pathogenic causes of GC, and cancer progression and metastasis are a process of multiple oncogenes [33]. Studies have also confirmed that c-Met interacts with multiple molecules in promoting cancer progression [34–36]. The results of these studies may also be caused by the interaction between c-Met and downstream carcinogens of *H. pylori* infection in the progression of GC. However, the occurrence and progression of GC caused by *H. pylori* infection is also the result of the action of multiple oncogenes. Therefore, the correlation between *H. pylori* infection and c-Met high expression requires more studies in the future to verify and explore its molecular mechanism.

As is known to all, AFP, CEA, CA199, CA153, CA125, and CA50 are common tumor markers of the digestive system. Exploring the correlation between these markers and c-Met may provide a new idea for optimizing c-Met targeting therapy strategies for GC. This study found that the correlation test between c-Met and serum CA125 level of patients was statistically significant, showing a positive correlation. Thus, it is possible to predict the expression of c-Met in tumors by detecting preoperative CA125 levels better to guide postoperative monitoring and prognosis assessment of patients. Recently, Hu et al. [37] conducted a study on CA125 and its prognosis in GC patients undergoing neoadjuvant chemotherapy and found that the level of CA125 before neoadjuvant chemotherapy was correlated with the prognosis of patients. The OS after chemotherapy decreased with the increase of CA125 levels. The study suggests that patients with serum CA125 normalization after neoadjuvant chemotherapy may benefit from survival. In addition, Zhou et al. [38] explored the relationship between serum CA19-9 and CA125 levels and HER2 expression in patients with GC and confirmed their correlation with the risk of recurrence and metastasis. The results showed that the recurrence and metastasis of GC patients with high levels of serum CA19-9, CA125, and tumor tissue-positive HER2 were significantly higher than those in the control group. Nevertheless, there was no correlation between serum CA19-9, CA125, and HER2-positive expression. These studies confirmed the correlation between serum CA125 level and prognosis of GC patients and the possibility of the correlation theory between c-Met and CA125. This finding is expected to help clinicians assess the role of c-Met in GC progression by monitoring the peripheral serum CA125 and thus better guide clinicians to choose c-Met inhibitors or c-Met-CAR-T cells therapy. Therefore, it provides ideas for targeting c-Met to treat GC and other solid tumors.

This study revealed that patients with high c-Met expression had a higher clinicopathological stage and a higher likelihood of tumor metastasis. High c-Met expression was also found to be associated with poor 5-year OS. In order to research the function of c-Met in different clinicopathological stages, subgroup analysis was also conducted according to the different clinicopathological stages of the tumor. The results demonstrated that the high expression of c-Met in stages I–II was associated with poor 5-year OS, while there was no correlation in stage III–IV patients. Zhang et al. and Yang et al. [39, 40] also found that the high expression of c-Met correlated with poor prognosis in GC patients, and the results were consistent with our study. However, their research on the patient was not for a more detailed analysis. Our research innovatively stratifies patients based on clinical-pathological staging. The results show that the c-Met at stage I–II tumor tissue plays a more critical role in promoting tumor proliferation and metastasis, while this effect in stage III–IV perhaps be weakened by other molecular mechanisms of cancer. After all, the molecular mechanism and regulation of promoting tumor proliferation and metastasis in advanced cancer are more complex. These studies suggest that inhibitors and mAb of c-Met may achieve more significant benefits in patients with early-stage GC.

Multivariate Cox regression analyses were performed to investigate further the risk factors associated with 5-year survival after surgery. The results revealed that age, clinicopathological stage, high expression of c-Met, and preoperative serum AFP might be independent risk factors for 5-year survival. Tobias Jagomast et al. [41] studied the prognostic value of c-Met in patients undergoing radical gastrectomy in Canada. The results demonstrated that c-Met high expression was correlative with poor OS. Multivariate analysis showed that the co-expression of EGFR and c-Met was an independent risk factor for postoperative survival of GC. However, Pereira et al. [42] recently reported that c-Met was associated with postoperative survival but not an independent risk factor for prognosis. These studies confirmed the role of c-Met in the progression of GC and demonstrated the important role of the interaction between EGFR and c-Met in GC progression. Therefore, this molecular interaction may account for the negative or positive results of c-Met being an independent risk factor for GC.

In addition, our study found that increased preoperative AFP may be an independent risk factor for postoperative survival of GC in our included population. Xu et al. [43] conducted a meta-analysis on the effect of serum AFP level on prognosis in patients with GC before treatment. Thirteen studies involving 9099 patients with GC were entered into the analysis. The results revealed that a high serum AFP level before treatment correlated with poor prognosis in GC patients. The above studies are consistent with our conclusions, suggesting that serum AFP level before treatment can act as a prognostic indicator of GC patients, and AFP can be used to assess the disease condition and prognosis of GC patients. However, AFP is a specific tumor marker of liver cancer, and its serum expression level in GC patients may be significantly lower than that in liver cancer patients. Therefore, more studies are needed to confirm whether AFP can be used as a specific tumor marker for GC to guide clinical practice.

This study also has some limitations. On the one hand, the sample size included in the study is limited, resulting in bias. Secondly, there is a lack of clinical data to monitor postoperative serum tumor markers in patients, leading to the failure of the correlation study between the above indicators and c-Met. However, the pathological data, clinical indicators, and preoperative serum tumor markers of patients in this study were relatively complete. Therefore, the conclusion of this study is detailed and reliable.

## Conclusions

In conclusion, this study describes the expression of c-Met in patients with GC and its correlation with prognosis. High expression of c-Met was associated with poor 5-year OS, especially in patients with clinicopathological stages I–II, and was an independent risk factor for postoperative survival in patients with GC. Meanwhile, the study found a positive correlation between the expression of c-Met in GC and the preoperative serum CA125 of patients. These findings have important clinical significance because they can guide the selection of patients with the appropriate pathological stage for the treatment of GC by targeting c-Met and better guide the postoperative monitoring and prognosis evaluation of patients with high c-Met expression by detecting the preoperative CA125 level of patients. It also confirms the importance of targeting c-Met therapy combined with its interacting molecular inhibitors in patients with advanced GC.

## Data Availability

The datasets used and/or analyzed during the current study are available from the corresponding authors on reasonable request.

## Abbreviations

c-Met: c-mesenchymal-epithelial transformation factor
HGF: Hepatocyte growth factor
GC: Gastric cancer
H-score: Histochemistry score
AFP: Alpha-fetoprotein
CEA: Carcinoma embryonic antigen
RON: Recepteur d ‘Origine Nantais
*H. pylori*: *Helicobacter pylori*
OS: Overall survival
HR: Hazard ratio
CI: Confidence interval
NSCLC: Non-small cell lung cancer
